# The geometry of interpersonal synchrony in human dance

**DOI:** 10.1016/j.cub.2024.05.055

**Published:** 2024-07-08

**Authors:** Félix Bigand, Roberta Bianco, Sara F. Abalde, Giacomo Novembre

**Affiliations:** 1 Neuroscience of Perception & Action Lab, Italian Institute of Technology, Viale Regina Elena 291, 00161 Rome, Italy; 2 The Open University Affiliated Research Centre, Istituto Italiano di Tecnologia, Genova, Italy

**Keywords:** dance, music, synchrony, social interaction, space, kinematics, 3D motion capture, component analysis, bounce, dance moves

## Abstract

Collective synchronized behavior has powerful social-communicative functions observed across several animal taxa.^1^^,^^2^^,^^3^^,^^4^^,^^5^^,^^6^^,^^7^ Operationally, synchronized behavior can be explained by individuals responding to shared external cues (e.g., light, sound, or food) as well as by inter-individual adaptation.^3^^,^^8^^,^^9^^,^^10^^,^^11^ We contrasted these accounts in the context of a universal human practice—collective dance—by recording full-body kinematics from dyads of laypersons freely dancing to music in a “silent disco” setting. We orthogonally manipulated musical input (whether participants were dancing to the same, synchronous music) and visual contact (whether participants could see their dancing partner). Using a data-driven method, we decomposed full-body kinematics of 70 participants into 15 principal movement patterns, reminiscent of common dance moves, explaining over 95% of kinematic variance. We find that both music and partners drive synchrony, but through distinct dance moves. This leads to distinct kinds of synchrony that occur in parallel by virtue of a geometric organization: anteroposterior movements such as head bobs synchronize through music, while hand gestures and full-body lateral movements synchronize through visual contact. One specific dance move—vertical bounce—emerged as a supramodal pacesetter of coordination, synchronizing through both music and visual contact, and at the pace of the musical beat. These findings reveal that synchrony in human dance is independently supported by shared musical input and inter-individual adaptation. The independence between these drivers of synchrony hinges on a geometric organization, enabling dancers to synchronize to music and partners simultaneously by allocating distinct synchronies to distinct spatial axes and body parts.

## Highlights

• Human dyadic dance involves distinct types of interpersonal synchronies
• Simultaneous music- and partner-driven synchronies unfold independently
• This independence relies on a spatial, geometric organization
• Bounce movements operate as a supramodal pacesetter of synchronization

## Graphical abstract

**Figure:**
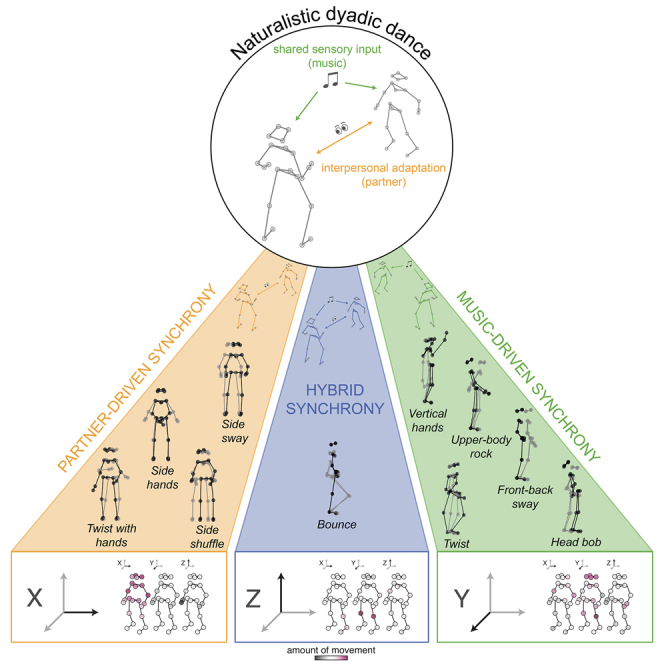

## Teaser

Synchrony in human dyadic dance can stem from shared sensory input (music) as well as from interpersonal adaptation (partner). Breaking complex kinematics into dance moves, Bigand et al. show that music- and partner-driven synchronies are orthogonal, even when they occur simultaneously, and that their independence relies on a geometric organization.

## Results

Social synchrony is widely observed across many animal taxa: from congregations of fireflies or crickets^2^^,^^3^ to flocks,^1^^,^^4^ fish schools,^1^^,^^5^ and several mammals.^6^^,^^7^ Multiple non-exclusive explanations have been put forward for the emergence of such collective group behaviors. Some accounts only require individuals to respond to common external cues (e.g., light, sound, or food), while others also imply inter-individual adaptation acting above and beyond the mere presence of a shared sensory input.^3^^,^^8^^,^^9^^,^^10^^,^^11^

**Figure 1:**
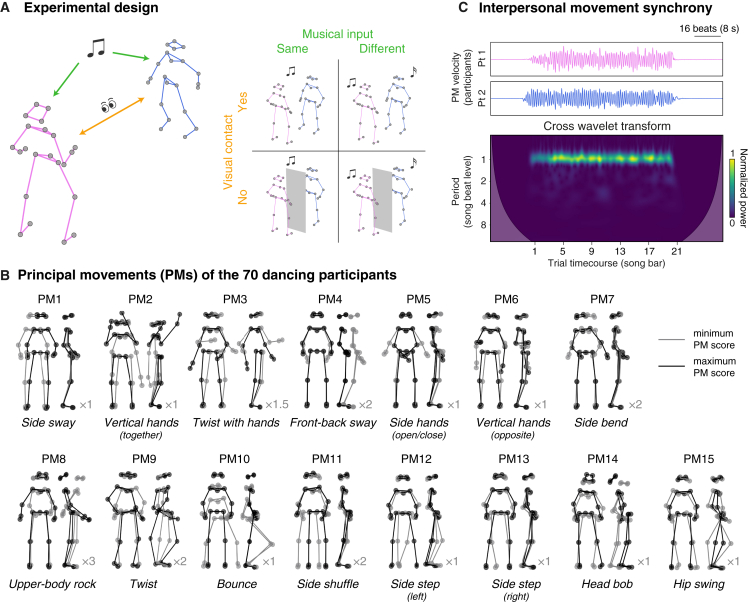
Experimental design and analytical methods (A) Experimental design. We tracked full-body kinematics from dyads of participants freely dancing to music. Synchrony in dyadic dance might only originate from listening to the same music (shared auditory input, green), but it can also emerge from interpersonal movement adaptation (orange). To study the interplay between music-driven and partner-driven synchrony, we manipulated musical input (whether participants listened to the same or different music, presented through earphones) and visual contact (whether participants could see or not see each other) in a 2 × 2 factorial design. (B) Extracted principal dance movements. Using principal-component analysis, the complex and naturalistic dance movements were decomposed into a set of one-dimensional uncorrelated components, which we refer to as principal movements (PMs). The first 15 PMs, taken together, explained more than 95% of the whole kinematic variance. The data associated with the PMs are illustrated by showing the two most different body postures (min and max of the PM score, in gray and black, respectively) from both frontal and sagittal perspectives (STAR Methods; Equation 2). The reader should interpret the PM as the kinematic displacement necessary to shift from one body posture (e.g., gray) to the other (e.g., black) (Video S1). For clarity, the PMs are plotted with different levels of exaggeration (i.e., the min and max were amplified by a factor—illustrated in the image next to each PM). PMs were reminiscent of common dance moves (spelled out in italics). A supplementary analysis assessed that quantity of movement was comparable across conditions, with the notable exception of PM10 (bounce; Figure S1). (C) Measure of interpersonal movement synchrony. Representative data (single trial, PM10) are displayed. Top: two time series (from two participants forming a dyad) representing velocity of the PM. Bottom: velocity time series are used to compute synchrony using cross-wavelet transform. Synchrony estimates are computed over distinct time points (throughout the time course of each song/trial, x axis) and periods (relative to the metrical structure of the music, i.e., beat levels, y axis). See also Figure S1.

Collective dance—an ancient and universally observed human behavior^12^^,^^13^^,^^14^—could be explained by either account. Humans spontaneously entrain to rhythmic auditory patterns that are typical of music^15^^,^^16^ as well as to body movements exhibited by a partner^17^^,^^18^^,^^19^^,^^20^^,^^21^^,^^22^ (Figure 1A, left). In complex and realistic settings, these two attractors (i.e., music and partners) might be operating at the same time, potentially causing mutual reinforcement^23^^,^^24^^,^^25^ but also competition.^26^^,^^27^

To study the interplay between music-driven and partner-driven social synchrony, we tracked full-body kinematics from dyads of human participants freely dancing to music (refrains of famous songs adapted for this study) in a “silent disco” setting. We manipulated music-driven synchrony by either playing the same (synchronous) or different (asynchronous) music to the two participants forming a dyad. Partner-driven synchrony was manipulated by enabling or disabling visual contact within the dyad (Figure 1A, right).

Using a fully data-driven method (STAR Methods), we were able to break down complex naturalistic kinematics into 15 one-dimensional principal movements (PMs) explaining over 95% of kinematic variance. The PMs were reminiscent of common “dance moves,” such as body sway, twist, upper-body side bend and rock, bounce, side displacement, head bob, hip swing, and hand movements (Figure 1B; Video S1).

Video S1. The principal (dance) movements, related to Figure 1 Video showing original movement data (left) and their decomposition into 15 principal movements explaining >95% of the kinematics variance (right). Representative data are displayed (excerpt from a single trial, corresponding to when participants listened to the full refrain of the song). For the sake of clarity, the PMs are animated with different levels of exaggeration (i.e. the PM scores were amplified by a factor of 1.5 (PM3), 2 (PM4,7,9,11,15), 3 (PM8), or not amplified (all other PMs)). The PMs are reminiscent of common dance moves (spelled out in italics).

**Figure 2:**
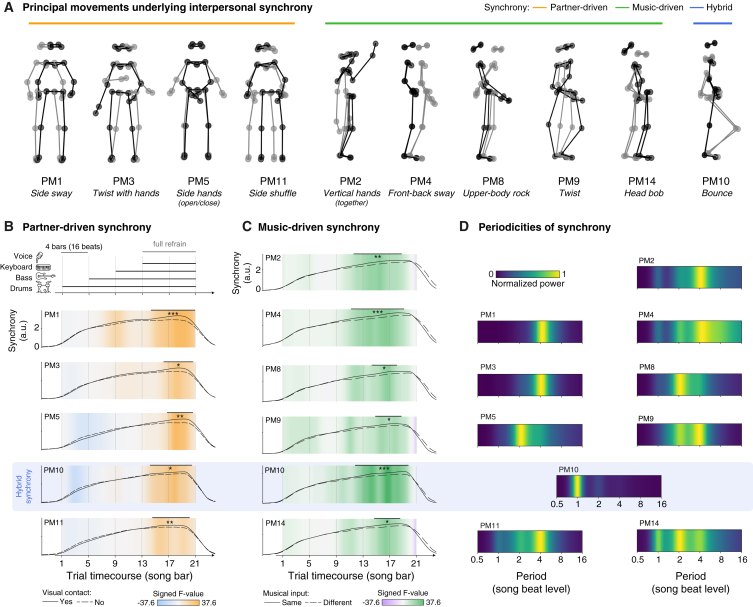
Characterization of the PMs underlying interpersonal synchrony (A) PMs that became more synchronized as a function of visual contact (partner-driven), shared musical input (music-driven), or both (hybrid) (see also Video S2). (B) Partner-driven synchrony. Continuous and dashed waveforms index the time course of interpersonal synchrony while participants were either able or not able to see each other, respectively. The colored background indexes the statistical difference between these two conditions (F values, signed by the difference between the two conditions, following cluster-based permutation [across time] and Bonferroni correction [across PMs]). Overlined sections indicate significant clusters (^∗∗∗^*p*_bonf_ < 0.001, ^∗∗^*p*_bonf_ < 0.01, ^∗^*p*_bonf_ < 0.05, cluster-corrected). (C) Same as (B), but referring to music-driven synchrony. Continuous and dashed waveforms index the time course of interpersonal synchrony while participants were either listening to the same or different music, respectively. Note that synchrony of only PM10 increased as a function of both visual contact and shared musical input (hybrid). (D) Periodicities of synchrony, relative to the 10 PMs presented in (A)–(C). Periodicities were computed by collapsing cross-wavelet power spectra over time and indicate the pace at which PMs synchronized across participants (relative to the beat levels of the music). See also Figure S2.

**Figure 3:**
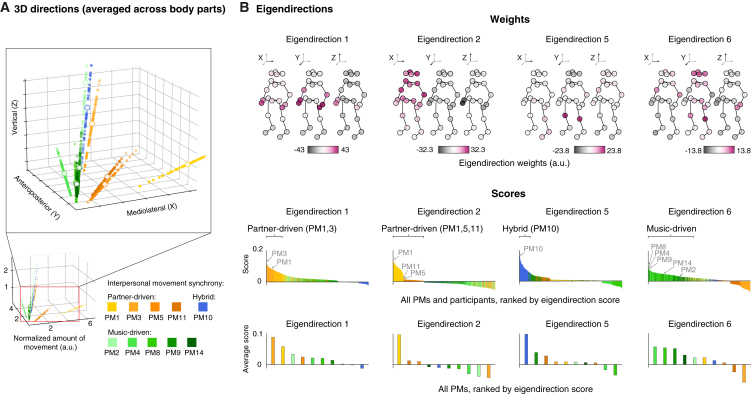
Geometry of interpersonal movement synchrony (A) 3D directions of the PMs underlying partner-driven, music-driven, and hybrid synchrony (averaged across body parts) (see also Video S2). The small, colored dots represent the main 3D directions of movement of the 10 identified PMs (Figure 2A) for each participant. Large white dots with colored contours represent the average directions in 3D space, across participants. For clarity, data are shown both in zoomed-in (top inlet) and full (bottom) frames. (B) Eigendirections. Top: weights in 3D body-part coordinates, mean centered. Large positive and negative weights indicate a relatively high and low amount of movement, respectively. Bottom: scores for all PMs and participants, as well as scores averaged across participants, ranked in descending order. Eigendirections 1 and 2 selectively show high scores for PMs characteristic of partner-driven synchrony. Eigendirection 5 selectively shows high scores for the PM characteristic of hybrid synchrony. Eigendirection 6 selectively shows high scores for PMs characteristic of music-driven synchrony. See also Figures S3 and S4.

Video S2. Spatially distinguishable movements underlying partner-driven, music-driven, and hybrid interpersonal movement synchrony, related to Figures 2 and 3 Representative video (same excerpt as in Video S1) showing original movement data (left) and extracted principal dance movements synchronizing either with the partner, the music or both (right). The principal dance movements (bottom) are linearly combined to visualize these three classes of movements (top).

Examining how these PMs synchronized interpersonally (Figure 1C), we demonstrate that music-driven and partner-driven synchronies are orthogonal, even when they occur simultaneously, and that their independence relies on a spatial organization.

### Orthogonality of music- and partner-driven interpersonal movement synchrony

We assessed whether and which specific PMs synchronized interpersonally, either through listening to the same music and/or through visual contact with the partner (Figures 2A–2C). Generally, across all PMs, interpersonal synchrony grew throughout the course of a song, each featuring four instruments (drums, bass, keyboard, and vocal melody) joining in incrementally until the “full refrain” of the song was played and repeated twice (Figure 2B, top). It was during this phase of the song, when all instruments were played simultaneously, that synchrony was highest and notably different across conditions (Figures 2B and 2C).

Statistics indicated that boosting auditory coupling through shared musical input, or visual coupling through visual contact, enhanced interpersonal synchrony of *distinct* PMs (Figure 2A). Synchrony of these PMs was enhanced either by listening to the same music (music-driven) or by seeing the partner (partner-driven). Only synchrony of one PM—bounce—was enhanced both by listening to the same music and by seeing the partner, but still independently (hybrid; Figures 2 and S2). Indeed, we found no evidence of an interaction between musical input and visual contact, and this was so for all PMs.

These results indicate that musical input and visual contact had fully orthogonal, i.e., independent effects on synchrony: even when participants were presented with non-synchronous music, some PMs would still synchronize with the movements of the dancing partner, just as when participants were presented with synchronous music. Conversely, another group of PMs would keep synchrony with music, even if the partner was visible and moving at a different pace.

### Geometric organization of interpersonal movement synchrony

Synchrony of specific PMs was driven by the music, the partner, or both (hybrid). Could these classes of PMs be distinguished based on their temporal or spatial properties? Visual inspection of the PMs suggested that these three classes of movements displayed comparable temporal periodicities (Figure 2D) but somewhat different spatial properties (Figure 2A). Specifically, while partner-driven synchrony appeared to rely on lateral movements, music-driven synchrony seemed to recruit anteroposterior movements, and hybrid synchrony was selectively associated with vertical bounce. This suggests (while not yet proving) that interpersonal synchrony in dyadic dance might be “geometrically” organized.

To formally test this hypothesis, we quantified the spatial properties of the 10 PMs (identified in the previous analysis; Figure 2A), namely where participants moved to achieve music-driven, partner-driven, and hybrid synchrony. In the first analysis (Figure 3A), we computed the main three-dimensional (3D) axes along which participants moved: the main direction of movement was averaged across body parts, separately for each PM. Supporting the hypothesis of geometric organization of synchrony, we found that the main 3D axis associated with partner-driven synchrony was lateral, while those associated with music-driven and hybrid synchrony were anteroposterior and vertical, respectively (Figure 3A).

**Figure 4:**
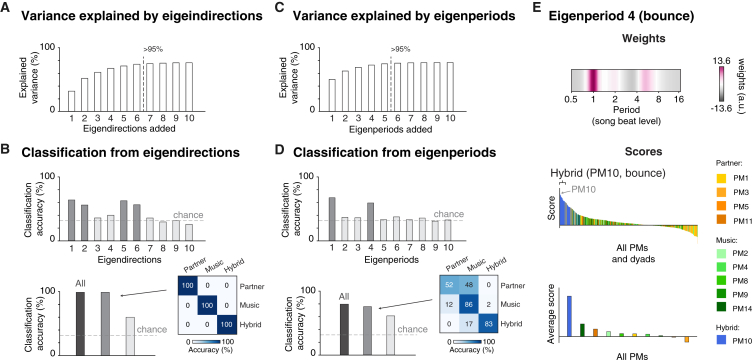
Geometric—but not temporal—organization underlines the independence between music- and partner-driven synchrony (A) Variance explained by eigendirections. The first six eigendirections explained more than 95% of the spatial variance across PMs and participants. (B) Accuracy of individual (top) or grouped (bottom) eigendirections in classifying PMs associated with music-driven, partner-driven, and hybrid synchrony. Top: the most important eigendirections for classification were eigendirections 1, 2, 5, and 6 (note that their classification accuracy is above chance). This is in line with how eigendirection scores were distributed over the three synchrony categories (Figures S4A and S4B). Bottom: eigendirections 1, 2, 5, and 6 (taken together) were sufficient to predict synchrony category with 100% accuracy, similar to a full model, including all first 10 eigendirections (A). (C) Same as (A), but referring to eigenperiods. The first five eigenperiods explained more than 95% of the temporal periodicity variance across PMs and dyads. (D) Same as (B), but referring to eigenperiods. Top: the most important eigenperiods for classification were eigenperiods 1 and 4. This is in line with how eigenperiod scores are distributed over the three synchrony categories (Figures S4C and S4D). Eigenperiods 1 and 4 (taken together) were not sufficient to predict synchrony category as accurately as a full model, including all first 10 eigenperiods (C). Notably, the model comprising only eigenperiods 1 and 4 confused 48% of the data associated with music-driven synchrony with partner-driven synchrony. (E) Eigenperiod characteristic of hybrid synchrony (i.e., interpersonal synchrony of bounce). Top: weights in period coordinates, mean centered. Large positive and negative weights indicate a relatively high and low amount of synchrony, respectively. Eigenperiod 4 primarily captured a period matching the musical beat. Bottom: scores for PMs and dyads, as well as scores averaged across dyads, ranked in descending order. Eigenperiod 4 primarily shows high scores for bounce (PM10), characteristic of hybrid synchrony. See also Figures S3 and S4.

Next, we tested whether this putative geometry of synchrony could emerge also from a fully data-driven analysis, including all body parts (note that the aforementioned analysis did not differentiate body parts). To do so, we used a second-layer principal-component analysis (PCA) that extracted “eigendirections” (Figure 3B), i.e., primary directions of movement that generalized across PMs^28^^,^^29^^,^^30^ (see STAR Methods and Figure S3 for details). This analysis yielded six eigendirections that explained >95% of the variance (Figure 4A). Notably, four of these eigendirections captured the cross-PM spatial patterns that were characteristic of partner-driven synchrony (eigendirections 1 and 2), music-driven synchrony (eigendirection 6), and hybrid synchrony (eigendirection 5) (see scores in Figures 3B and S4A). This was confirmed by a classification model demonstrating that the synchrony category could be predicted with 100% accuracy when only relying on these four eigendirections (Figure 4B). These results are remarkable considering that no information about synchrony category was used in the second-layer PCA.

The latter analysis also offered insights about the specific body parts underlying synchrony categories (see weights in Figure 3B). First, while partner-driven synchrony primarily relied on full-body lateral movements (eigendirection 2), it also entailed hand movements that synchronized freely across all 3D axes (eigendirection 1). Second, music-driven synchrony relied on anteroposterior movements across knees, trunk, and head, although the left hand and both elbows were also slightly synchronized along vertical and lateral axes (eigendirection 6). Finally, and fully in line with what we observed above, hybrid synchrony was achieved through a vertical movement, i.e., bounce (eigendirection 5).

Complementing the previous set of results indicating that music-driven and partner-driven synchronies can occur simultaneously and orthogonally, this set of data-driven analyses demonstrated that such orthogonality hinges on a geometric organization of synchrony. Such organization distributes distinct synchronies across distinct spatial axes and body parts, permitting music-driven and partner-driven synchronies to carry on independently and simultaneously.

### Timing alone is not sufficient to distinguish music-driven from partner-driven synchrony

The above analyses demonstrated that interpersonal synchrony in dyadic dance is geometrically organized: spatially distinct body movements synchronize through music listening or partner vision, independently. Does this spatial organization have an equivalent in the temporal domain? In other words: is it possible to infer whether synchrony of these distinct movements is achieved through music listening versus partner vision, by examining when, i.e., at which periodicity, the body moves?

Observing the periodicities of interpersonal synchrony—i.e., the pace at which PMs synchronized—movements with a pace either 2 or 4 times the musical beat seemed to contribute indistinctively to partner- and music-driven synchronies (Figure 2D). We formally tested the validity of this observation with a data-driven analysis mirroring the one described in the previous section: we extracted “eigenperiods” and tested whether they could predict synchrony categories. This analysis yielded five eigenperiods that explained >95% of the variance (Figure 4C). Notably, eigenperiods failed to capture any temporal pattern characteristic of music- or partner-driven synchronies (see classification accuracy in Figure 4D and eigenperiod scores in Figures S4C and S4D). Eigenperiod 1 captured shared temporal properties of only two of the four PMs underlying partner-driven synchrony, while eigenperiods 2, 3, and 5 were either PM specific or generally unspecific. This result indicates that *when* (unlike *where*) participants move does not reflect whether synchrony is established through music or partner vision. Hence, the independence between music- and partner-driven synchrony is achieved solely through a spatial, not temporal, organization.

Notably, eigenperiod 4 captured a specific pace—matching the musical beat—that was distinctively associated with hybrid synchrony (see weights in Figure 4E; see also Figure 2D). Furthermore, the classification model (relying on the only two informative eigenperiods: 1 and 4; Figure 4D) showed that 83% of the data associated with hybrid synchrony were correctly predicted as such, although occasionally (17%) confused with music-driven synchrony (Figure 4D).

### Uniqueness of bounce in mediating interpersonal synchrony

Three pieces of evidence from our study suggest that vertical bounce is somehow special. First, only bounce synchronized through both the music and the partner, albeit independently: only listening to the same music or only seeing the partner would boost synchrony just as much as when the two factors operate simultaneously (Figures 2B, 2C, and S2). Second, only bounce increased in magnitude (quantity of movement) when participants could see each other (Figure S1). Third, bounce is the only movement that synchronized at a specific temporal periodicity, notably matching the musical beat (Figures 2D and 4E). These results suggest that bounce might play a unique role in mediating interpersonal synchrony and will be further discussed below.

## Discussion

We examined the interplay between two factors contributing to the emergence of social synchrony in dyadic dance: shared sensory input (music) and interpersonal adaptation (partner) (Figure 1). We show that synchrony of specific dance moves is independently driven either by music or by the dance partner, resulting in a full dissociation between music-driven and partner-driven synchronies (Figure 2). This dissociation is enabled by a spatial organization, referred to as a “geometry” of interpersonal synchrony, which distributes distinct synchronies (music- or partner-driven) across distinct spatial axes and body parts (Figure 3). Further highlighting the importance of such spatial organization, our work also demonstrates that the independence between music- and partner-driven synchrony cannot be explained when only relying on temporal features (Figure 4). Together, these results demonstrate that social synchrony in human dyadic dance stems from simultaneous, independent, and spatially organized yet temporally indistinguishable types of synchronization.

Our results imply that group synchronized behavior should not be viewed as a unitary, indivisible phenomenon. Within the same dyad, multiple synchronization processes can occur simultaneously, exploiting either the presence of a shared sensory input, or interpersonal adaptation. The distinction between these facets of synchronization is often overlooked, even if it has been theoretically acknowledged in several fields, including ethology,^8^ psychology,^9^ musicology,^11^ and neuroscience.^10^ Here, by decomposing naturalistic kinematics into a discrete set of movement primitives, we were able to provide evidence for the independence of these distinct kinds of synchronization, even when they occur simultaneously: even when participants were dancing to non-synchronous music, some movements synchronized with the dancing partner, similar to when synchronous music was played. Conversely, other movements stayed in sync with/through the music, even if the partner was visible and moved at a different pace.

How can multiple synchronization processes unfold independently? We demonstrate that this can be achieved leveraging the geometric organization of interpersonal synchrony. Under ecologically valid conditions, such as when two individuals dance face-to-face, partner-driven synchrony relies on lateral movements, while music-driven synchrony depends on anteroposterior movements. This spatial organization could be partially explained by contextual constraints: participants are likely to produce visually salient cues more effectively through horizontal movements,^31^ while keeping anteroposterior movements relatively hidden from the view of their dancing partner. Such covertness facilitates synchronization to the music, allowing participants to maintain their individual pace without compromising mutual synchrony.^32^^,^^33^ Whether this spatial organization remains adaptable to changing environmental constraints is yet to be determined. In a less-common dyadic configuration, such as dancing side-by-side without physical contact, the geometry may self-organize differently, potentially leading to partner-driven synchrony to rely more on anteroposterior movements. Alternatively, or complementarily, it is possible that the geometry is dictated by rigid functional roles of specific body movements. For example, head bobs are known to support precise synchronization with music,^15^^,^^34^^,^^35^^,^^36^ perhaps even in non-human species^37^^,^^38^^,^^39^ (see PMs 8 and 14), while hand gestures mediate much social and communicative behavior^40^^,^^41^ (see eigendirection 1). Regardless, the geometric organization we described is sufficient to maintain music- and partner-driven synchronies independently, even though different morphologies may emerge under different contexts.

We also described a third kind of synchrony—hybrid synchrony—which was uniquely contributed by vertical bounce (PM10). Bounce was the only movement that synchronized interpersonally through both the music and the partner (but note that even in this case the two drivers of synchrony would not interact; see results and Figures 2A–2C and S2). Bounce was also the only movement that precisely synchronized to the musical beat (Figures 2D and 4E) and that was enhanced in magnitude when participants were seeing each other (Figure S1). Together, these results fit well with research examining beat synchronization, indeed showing that it can be effectively achieved through both visual and auditory modalities,^31^^,^^42^^,^^43^ and often through vertical movements.^16^^,^^44^^,^^45^^,^^46^^,^^47^ We suggest that vertical bounce could be seen as a supramodal pacesetter that individuals emphasize to achieve interpersonal synchrony. Tracing the physiological reasons why bounce might be so effective in signaling temporal information, we highlight the idea that bounce triggers several sensory feedback signals that might reinforce internal timekeeping and, in turn, interpersonal coordination.^48^^,^^49^ For instance, bounce causes fast movement of the head, leading to strong vestibular responses.^34^^,^^35^ It also exploits gravity and produces proprioceptive feedback arising from contact with the ground.^50^^,^^51^^,^^52^^,^^53^ Research in development might further reinforce our argument because bounce is a core component of locomotion,^28^ one of the first isochronous signals infants experience through maternal walking,^54^ and so far one of the few movements or postural (i.e., bipedal) contexts in which non-human primates synchronize movements interpersonally.^39^^,^^55^^,^^56^

“Synchrony” holds inherent temporal meaning. Yet examining (movement) temporal features alone, social synchrony might appear as a unitary, indivisible phenomenon, even when it is not. Spatial analyses of movement allowed us to dissect complex kinematics into distinct dance moves and to provide evidence for independent music- and partner-driven synchronization processes. Also relying on spatial analyses, we demonstrated that the independence between music- and partner-driven synchronies is enabled by a geometric organization whereby distinct synchronies are distributed across distinct spatial axes. We foresee that, as encouraged by our work, spatial analyses of collective behavior will reveal far-reaching insights into the organization of social synchrony and real-world collective behavior.

## STAR★Methods

### Key resources table

**Table:** 

REAGENT or RESOURCE	SOURCE	IDENTIFIER
**Deposited data**		

Kinematic data and musical stimuli	This paper	https://doi.org/10.48557/UR2GBG

**Software and algorithms**		

PLmocap	Bigand, PhD thesis^57^	https://github.com/felixbgd/PLmocap
Analysis code	This paper	https://github.com/felixbgd/geometry-IMS-dance

### Resource availability

#### Lead contact

Further information and requests for resources should be directed to and will be fulfilled by the lead contact, Félix Bigand (felix.bigand@iit.it).

#### Materials availability

All musical stimuli and metadata are publicly available at https://doi.org/10.48557/UR2GBG

#### Data and code availability

• Kinematic data have been deposited to IIT Dataverse and are publicly available as of the date of publication. Accession numbers are listed in the key resources table.
• All original code is publicly available on Github repositories. The links are listed in the key resources table.
• Any additional information required to reanalyze the data reported in this paper is available from the lead contact upon request.

### Experimental model and study participant details

A total of 80 participants, forming 40 dyads, were recruited (54 females; mean age: 26.15 years, SD: 6.43 years, 74 right-handed). We aimed to collect at least 28 dyads according to previous studies^17^^,^^58^ and based on an a priori power analysis (minimum N=28 to detect a medium-large effect size (Cohen’s f-value between 0.3 and 0.4)). To minimize inter-individual variability while maximizing generalizability, we recruited only laypersons (i.e. individuals without dance training), although we acknowledge that it would be interesting to compare individuals across different levels of expertise or cultural backgrounds. All pairs of participants were familiar with each other. All participants self-reported normal or corrected-to-normal vision, normal hearing, and no history of neurological disorders. The experimental procedures were in accordance with the Declaration of Helsinki and approved by “Comitato Etico Regionale della Liguria” (794/2021 - DB id 12093). All participants gave informed written consent to participate in the study and were compensated €25.

### Method details

#### Musical stimuli

Stimuli were eight musical songs (mean duration: 39.8 s, SD: 1.95 s). They consisted of rearrangements of well-known danceable songs taken from electronic dance music and disco-funk genres (Table S1). All songs were rearranged using the same four musical instruments: drums, bass, keyboards, and violin (which substituted the vocal melody). All songs were in a 4/4 meter and lasted 20 bars. For each instrument, the 4-bar loop of the original refrain was recreated by author FB and a professional composer (Raoul Tchoï), first transcribing it in MIDI and then synthesizing it using MIDI instruments in Logic Pro X (Apple, Inc.). The rearranged songs were then systematically structured by repeating the 4-bar loops five times and sequentially adding each instrument to the musical scene, in the following order: (1) drums, (2) bass, (3) keyboards, (4) voice, (5) voice *bis* (i.e. the loop with all instruments was repeated twice). Loudness was controlled across songs within a range of 1.5 LUFS (a measure that considers the sensitivity of the human auditory system across frequencies). Songs were played to dyads through two separate channels (one for each participant), using two pairs of earphones (Etymotic ER3C) connected to a multi-channel audio interface (RME Fireface UC).

Each trial included the presentation of one song flanked with a fast-rising tone (rise time 5 ms, fall time 30 ms, frequency 494 Hz, duration 350 ms), 8-s silence before and 7-s silence after, according to the following structure: beep-silence-song-silence-beep. Trials presentation was controlled using Presentation software (Neurobehavioral systems). Triggers synchronized the kinematics recordings with trial start, song start, and trial end. They were sent via TTL pulses to the computer used for acquiring kinematic data (Nexus, Lock+; Vicon).

#### Design and procedure

Trials were presented according to a 2x2 within-dyad factorial design. Factors were visual contact (Yes, No) and musical input (Same, Different). We manipulated visual contact by placing (or not placing) a curtain in between the two participants. We manipulated musical input by presenting either the same song or different songs to the participants (through earphones). The tempo of the songs presented (simultaneously) to the two participants was either perfectly synchronous (same-music condition) or slightly asynchronous (different-music condition). To minimize inter-trial variability, the latter asynchrony was controlled to be constant across trials (i.e. 8.5% relative tempo difference across songs; Table S2). This was achieved by presenting participants with songs of different genres during the different-music condition (note that electronic dance music songs were overall faster than disco-funk songs, see Table S1).

The 32 trials were grouped into four blocks. Each block included eight trials (two trials per condition, and each trial comprising a distinct song). The blocks and trials were presented in random order, with the exception that we always presented pairs of subsequent yes-vision or no-vision trials to minimize the displacement of the curtain.

The experimental environment was described (to the participants) as a “silent disco”. Participants were told that they could be presented with the same music or different music, but the specific trial category was unbeknown to them. Participants were instructed to look towards the other, to enjoy the music, and to remain still during the silence periods (before and after the music). During the experiment, participants stood in front of each other, each on a 0.5x0.7m marked space separated from one another by 2.5 meters. Participants were told that they were allowed (but not required) to dance freely to the music while staying in their marked space and maintaining the head orientation (towards the partner) as constant as possible. Participants were neither allowed to speak nor to sing during trials.

#### Data acquisition

3D full-body kinematics were recorded using wearable markers (n=22 per participant, size=14 mm). Markers were placed on the following body parts (L = left, R = right, F = front, B = back): (1) LB Head, (2) LF Head, (3) RF Head, (4) RB Head, (5) Sternum, (6) L Shoulder, (7) R Shoulder, (8) L Elbow, (9) L Wrist, (10) L Hand, (11) R Elbow, (12) R Wrist, (13) R Hand, (14) Pelvis, (15) L Hip, (16) R Hip, (17) L Knee, (18) L Ankle, (19) L Foot, (20) R Knee, (21) R Ankle, (22) R Foot (Figure 1A). One supplementary marker was located asymmetrically across participants (i.e. on the left or right thigh). This marker was used by Nexus software to distinguish the two participants, but it was not included in the analysis. The movement of the markers was captured by eight optical motion capture cameras (Vicon system; sampling rate: 250 Hz). The cameras framed the participants from multiple viewpoints, ensuring that at least six cameras could frame each participant even during the no-vision trials (when the curtain was used to separate the two participants).

### Quantification and statistical analysis

#### Extracting principal dance movements

Marker trajectories were first corrected for swaps or mislabels using Nexus manual labelling tool (Vicon). Frequent and systematic marker swaps were automatically corrected using custom code written in Python. Remaining gaps in the marker trajectories were filled using Nexus automatic gap-filling pipelines. Finally, all trajectories were visualized using Nexus, and manually corrected if they did not match the aerial view video recording. Five out of the 40 recruited dyads were excluded from further analysis because of recording failure. All subsequent data processing was conducted in Python using custom code. The marker trajectories consisted of 3D positions (along x, y, z axes) associated with each of the 22 body parts, yielding 66-dimensional temporally resolved posture vectors. These data were down-sampled to 25 Hz and trimmed according to song length.

We used Principal Component Analysis (PCA) to decompose the kinematic data into a set of Principal Movements (PMs) generalizable across trials, conditions and participants.^28^^,^^59^^,^^60^^,^^61^ First, to ensure that PCA would not capture anthropometric differences but only common movements across participants, posture vectors were demeaned and standardized across time, separately for each trial. Specifically, the mean posture vector (averaged across time) was subtracted from posture vectors of each frame. Next, these demeaned posture vectors were divided by a global measure of standard deviation across time (i.e. computed combining all body parts, to preserve between-body-part differences in variance). This normalization allowed pooling (i.e. concatenating) the posture vectors **p**(t) of all participants and trials into a data matrix **P** (2,177,912 frames × 66 [22 body parts along the 3 axes]), while ensuring an equal contribution of all participants and trials to the pooled matrix variance. PMs were derived from the eigenvectors **w** and their respective scores c(t) in the PCA applied to **P**, each of which reflects covarying marker trajectories:

(Equation 1) $$p\left(t\right)=\sum_{i}c_{i}\left(t\right)w_{i}$$

where **p**(t) is the original posture vector at time t (1 × 66), c_i_(t) is the i^th^ PM score at time t and **w**_**i**_ is the i^th^ PM weight vector, or eigenvector (1 × 66).

Note that PMs can thus be visualized (as in Figure 1B) by reconstructing the posture vector only using the i^th^ PM scores and weight vector:

(Equation 2) $$\text{PM}\left(t\right)=c_{i}\left(t\right)w_{i}$$

The first 15 PMs – accounting for more than 95% of the kinematic variance – were retained for further analyses.^60^^,^^61^ PCA was used to extract PMs based on previous evidence suggesting that such linear combinations accurately model the neural control of complex movements,^59^^,^^62^ and even the specific coordination patterns of dance.^16^^,^^63^ Further, we found that the variance explained by our 15 PMs was similar to that explained by non-linear components extracted using an auto-encoder (95.6% vs. 96.5%, respectively), confirming recent evidence that PCA can model high-dimensional kinematic data as efficiently as non-linear reduction techniques.^64^^,^^65^ The auto-encoder was trained using cross-validation (20% split) for 10,000 iterations (except if the validation loss would stop improving before). Its architecture was similar to the original one proposed by Kramer (1991)^66^ with a non-linear activation function (here hyperbolic tangent) for the first and third hidden layers, and a linear activation function for the middle one (bottleneck).

Power spectral density of the PM timeseries was computed using Welch’s method^67^ (window size 2 s, overlap 75%), and revealed that most power resided below 4 Hz. Therefore, PM timeseries were low-pass filtered below 6 Hz using a Butterworth filter (second-order, zero-phase) to increase signal-to-noise ratio. An example of a trial decomposed into 15 PMs can be seen in Video S1.

#### Music- and partner-driven interpersonal movement synchrony

The PM timeseries were padded by adding 3-bar-long segments of data anticipating and following presentation of the songs. These padded timeseries were obtained by multiplying the original extended data (including the 3 bars before and after) by the PM weight matrix.

The PM timeseries were turned into velocity timeseries by computing their first derivative. Next, pairs of velocity timeseries (one for each of the participants forming a dyad) were entered into a Cross-Wavelet (CW) transform, which yielded PM-specific estimates of interpersonal movement synchrony. The CW transform provided indices of period locking between participants’ PMs, across distinct timepoints and period bins.^68^^,^^69^ The CW transforms were calculated using the Morlet wavelet (w_0_ = 6), with 83 log-spaced periods between 0.5 to 16 times the song-specific musical beat. In the different-music trials, when participants listened to different songs, two CW transforms were computed in relation to the musical beat of each song and their results were averaged. Furthermore, to compare trials associated with songs of different length and tempo, trial-specific CW power spectra were down-sampled across time to fit the temporal scale of the shortest song. This resulted in the musical beat being the unit of both dimensions of the CW spectra, i.e. period and time. In other words, synchrony period equaling 1 implies that PMs synchronized at the same pace of the musical beat, while higher periods such as 2 or 4 imply that PMs synchronized twice or four times slower, respectively (Figure 1C). Likewise, the temporal unfolding of synchrony could reveal enhancements of synchrony at specific moments of the music (e.g. after the entrance of a new instrument, namely every 16 musical beats/4 bars). Finally, synchrony indices were smoothed over time with a rolling average window of 3 bars (moving in steps of 1 frame).

Single-trial synchrony spectra were averaged across periods, and the resulting timeseries were averaged to yield one timeseries per condition, dyad, and PM. These averages were standardized across conditions, within each dyad and PM, and then entered into a 2x2 repeated-measures ANOVA with factors visual contact and musical input. ANOVAs were calculated separately for each time point and each PM, resulting in timeseries of F-values for every PM. To correct for multiple comparisons across the 15 PMs, p-values were Bonferroni-corrected. To correct for multiple comparisons across time points, we used non-parametric cluster-based permutation testing^70^^,^^71^ using MNE Python.^72^ We first identified clusters with at least two consecutive time points with p_bonf_<0.05, and then summed F-values within each cluster. Next, we used permutation testing across experimental conditions to generate a random distribution (10,000 permutations). This distribution was used to define a cluster significance threshold and only significant clusters (p_bonf_<0.05) were retained.

#### Identifying the spatial organization of interpersonal movement synchrony

##### Eigendirections

Significant ANOVA clusters were used to group PMs into three synchrony categories (see Figures 2 and S2): PMs associated with partner-driven synchrony (i.e. associated with a main effect of visual contact), music-driven synchrony (i.e. associated with a main effect of musical input) and hybrid synchrony (i.e. both main effects) (note that we did not find evidence of an interaction between visual contact and musical input; see Figure S2). In total, 10 distinct PMs fell into either of the three categories. Here, we employed a second-layer PCA to extract “eigendirections”, i.e. primary directions of movement that generalized across these 10 PMs of interest (Figure S3). The rationale behind this analysis was that PMs belonging to the same category might be spatially organized and therefore display common spatial properties.

PMs of every trial were first reconstructed in the high-dimensional space, yielding timeseries of 66-dimensional vectors (22 body parts along the 3 axes). Next, spatial maps of each PM (i.e. the movement magnitude of each body part along the 3 axes) were computed as the mean absolute value of velocity across time, separately for each trial and participant, yielding 66-dimensional vectors. For each participant, these single-trial vectors were averaged across trials and standardized across PMs, yielding one spatial map per participant and PM. These spatial maps were mean-centered (i.e. reflecting how much each body part moved, relatively to the average magnitude across body parts), and were stored in a data matrix **D** (22 body parts along the 3 axes × 10 PMs across the 70 participants). We conducted two analyses to investigate these spatial maps. First, we assessed the main 3D axis along which participants moved across PMs, by averaging each spatial map across body parts. Second, we used a data-driven method to unveil the presumed spatial organization of these PMs, along 3D axes and across body parts^28^^,^^29^^,^^30^ (Figure S3). We extracted primary spatial maps, “eigendirections”, that explained most of the spatial variance across PMs and participants. Each of these eigendirections potentially reflects a spatial property of movement associated with either partner-driven, music-driven, or hybrid synchrony. To do so, PCA was applied to **D** (22 body parts along the 3 axes × 10 PMs across the 70 participants) transforming **D** into a set of K components (“eigendirections”) maximizing the spatial variance:

(Equation 3) D≈UV

where **U** is the weight matrix for the K eigendirections with highest variance (22 body parts along the 3 axes × K eigendirections) and **V** is the eigendirection score matrix (K eigendirections × 10 PMs ^∗^ 70 participants). With PCA, **U** and **V** can be either negative or positive without changing the results, so the sign of the eigendirections was fixed so that the biggest eigendirection scores were always positive, to ease visualization.

##### Classification

We used a classification analysis to formally test whether specific eigendirections could reflect distinctive spatial properties of PMs associated with either partner-driven, music-driven, or hybrid synchrony. A ridge logistic regression model was trained to classify the synchrony category of each PM based on their eigendirection scores (see Figures 4A and 4B). Specifically, a binary model was fit for each category (i.e. the model learns to predict 1 for PMs of the target category, and 0 for all other PMs). Leave-one-participant-out cross-validation was used to assess how well the model could classify unseen data while controlling for overfitting. Cross-validation thus consisted of training the model on eigendirections of N-1 participants (N=70) and testing it on the left-out participant. This procedure was repeated N (70) times and performance was quantified as the average amount of correct classifications on the test data.

Prior work has demonstrated that principal components with highest variance are not necessarily the most critical ones for successful classification tasks.^30^ Therefore, not only did we identify a subset of eigendirections that explained most of the variance in the data, but we also inspected how eigendirections allowed the classifier to predict synchrony categories accurately, independently from the variance explained.

#### Temporal organization of interpersonal movement synchrony

We also investigated the temporal information of the 10 identified PMs (see Figure 2D), repeating the same procedure used with spatial information (i.e. PCA and classification, see Figures 4 and S4). The temporal profile of each PM (i.e. the periodicity at which the PM synchronized) was derived from the cross-wavelet spectrum averaged across time, for each trial and dyad. These temporal periodicity profiles consisted of 83 period bins, spanning kinematic periods from 0.5 to 16 times the musical beat (see Figure 2D). Next, as for the spatial maps, the temporal periodicities were (1) mean-centered (i.e. reflecting how each period exhibited synchrony, relatively to the average synchrony across periods), (2) combined across PMs and dyads in a data matrix **D**_**period**_ (83 periods × 10 PMs across 35 dyads), (3) transformed into a set of K_period_ eigenperiods, and (4) used to train a classifier to predict synchrony category of the PMs based on the eigenperiod scores. Leave-one-dyad-out cross-validation of the model allowed us to formally test whether specific eigenperiods reflect distinctive temporal properties of PMs associated with partner-driven, music-driven, and hybrid synchrony.

## Supplemental information

Document S1. Figures S1–S4 and Tables S1 and S2

Document S2. Article plus supplemental information
