# Supplementary material for: The geometry of interpersonal synchrony in human dance
Source: Curr Biol. 2024 Jul 8;34(13):3011–3019.e4. doi: 10.1016/j.cub.2024.05.055 (PMC11266842; doi:10.1016/j.cub.2024.05.055)
Supplement: Document S1. Figures S1–S4 and Tables S1 and S2 [file mmc1.pdf]

Current Biology, Volume *34*

Supplemental Information

The geometry of interpersonal synchrony in human dance

Félix Bigand, Roberta Bianco, Sara F. Abalde, and Giacomo Novembre

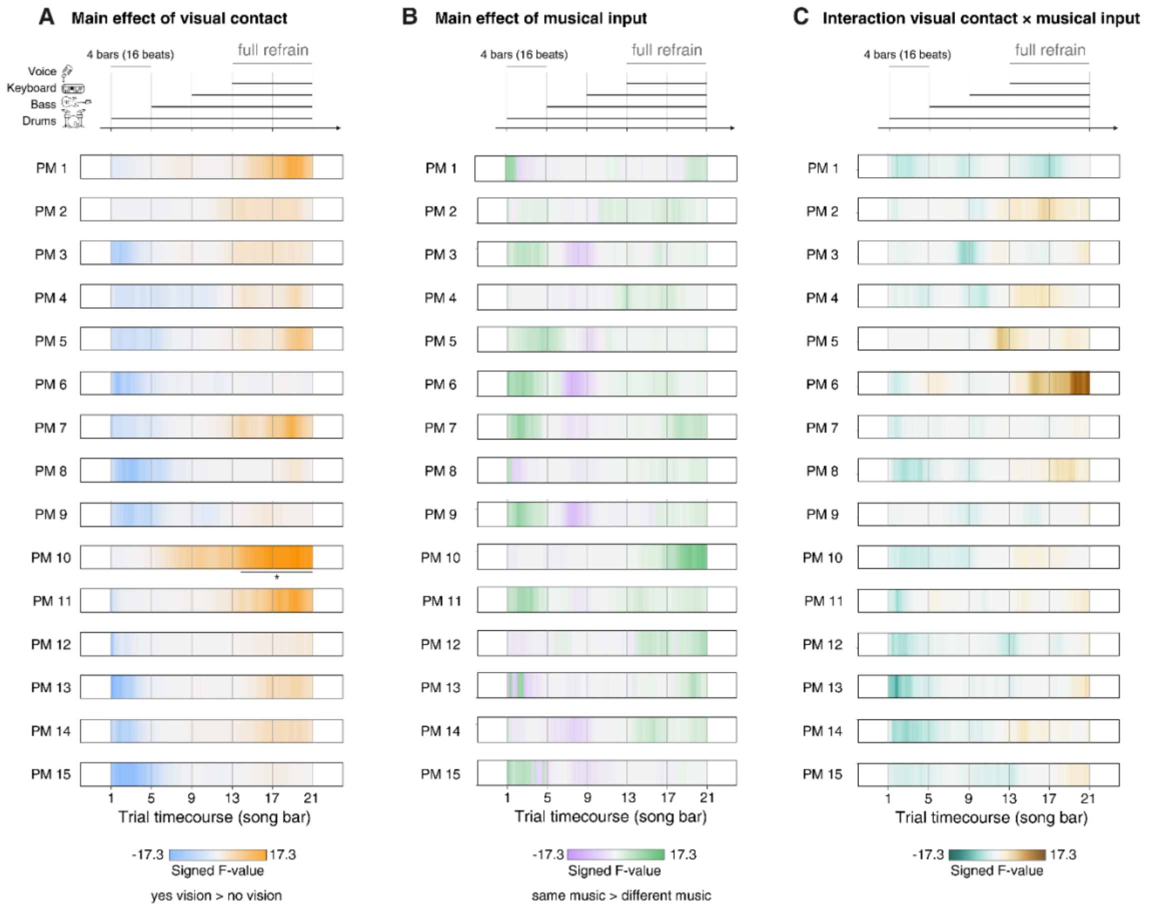

Figure S1. Effects of visual contact and musical input on quantity of movement. Related to Figure 1. (A) Main effect of visual contact. For each trial, quantity of movement was estimated by computing the average absolute value of each PM velocity, separately for each of the two participants forming a dyad, and then by summing the resulting values across the two participants (to meet the ANOVA's requirement of data samples independency). Prior to velocity computation, PM timeseries were destandardized (i.e. multiplied by the single-trial standard deviation computed across all markers) to retain cross-condition variance. The coloured background indexes the time-course of the statistical difference between the yes-vision and no-vision conditions (F-values, signed by the difference between the two conditions, following cluster-based permutation (across time) and Bonferroni correction (across PMs)). Underlined sections indicate significant clusters (***$p_{bonf}<0.001$, **$p_{bonf}<0.01$, *$p_{bonf}<0.05$, cluster-corrected). Note that quantity of movement of only PM10 increased as a function of visual contact. **(B)** Same as A, but referring to the main effect of musical input on quantity of movement. **(C)** Same as A and B, but referring to the interaction between visual contact and musical input.

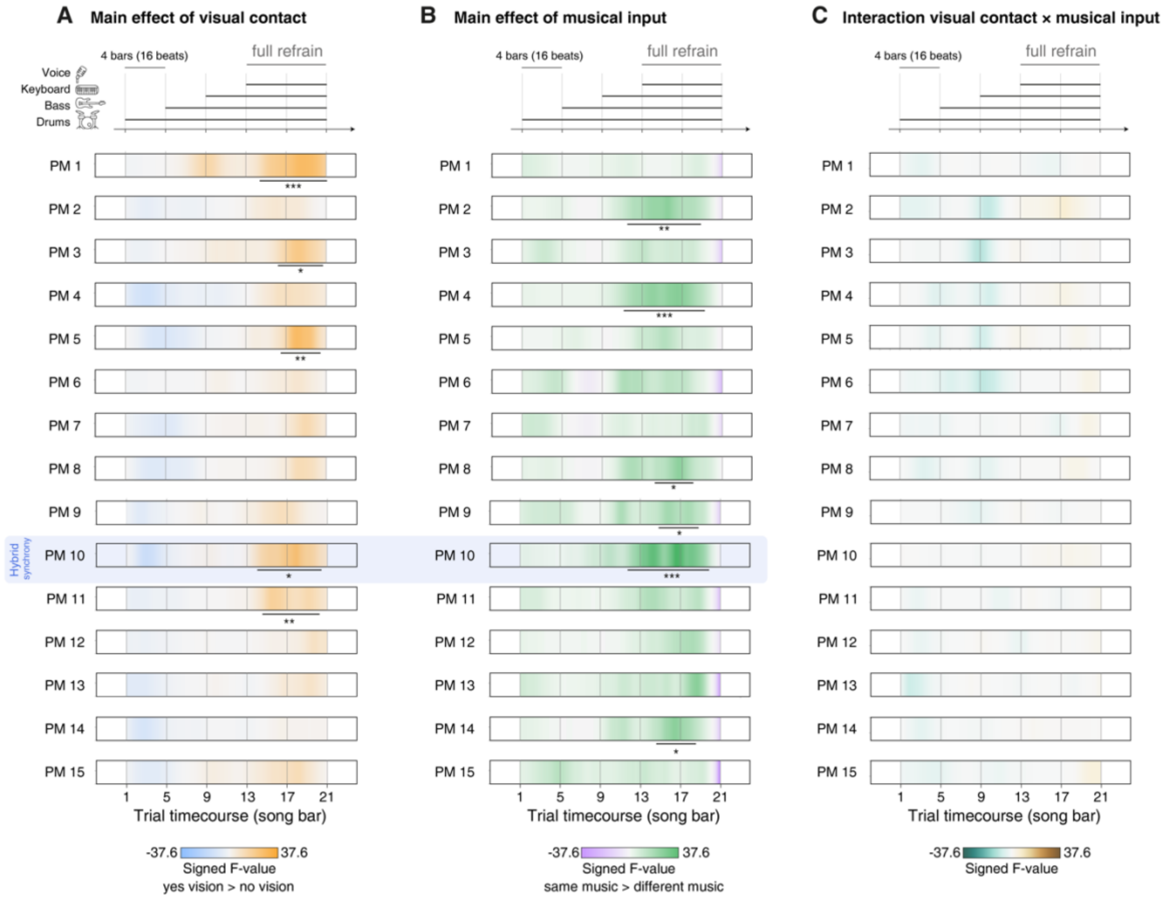

Figure S2. Effects of visual contact and musical input on interpersonal synchrony (all 15 Principal Movements (PMs)). Related to Figure 2. (A) Main effect of visual contact. The coloured background indexes the time-course of the statistical difference between the yes-vision and no-vision conditions (F-values, signed by the difference between the two conditions, following cluster-based permutation (across time) and Bonferroni correction (across PMs)). Underlined sections indicate significant clusters (***$p_{bonf}<0.001$, **$p_{bonf}<0.01$, *$p_{bonf}<0.05$, cluster-corrected). **(B)** Same as A, but referring to the main effect of musical input. Note that synchrony of only PM10 increased as a function of both visual contact and shared musical input. **(C)** Same as A and B, but referring to the interaction between visual contact and musical input. No PM was associated with an interaction between visual contact and musical input.

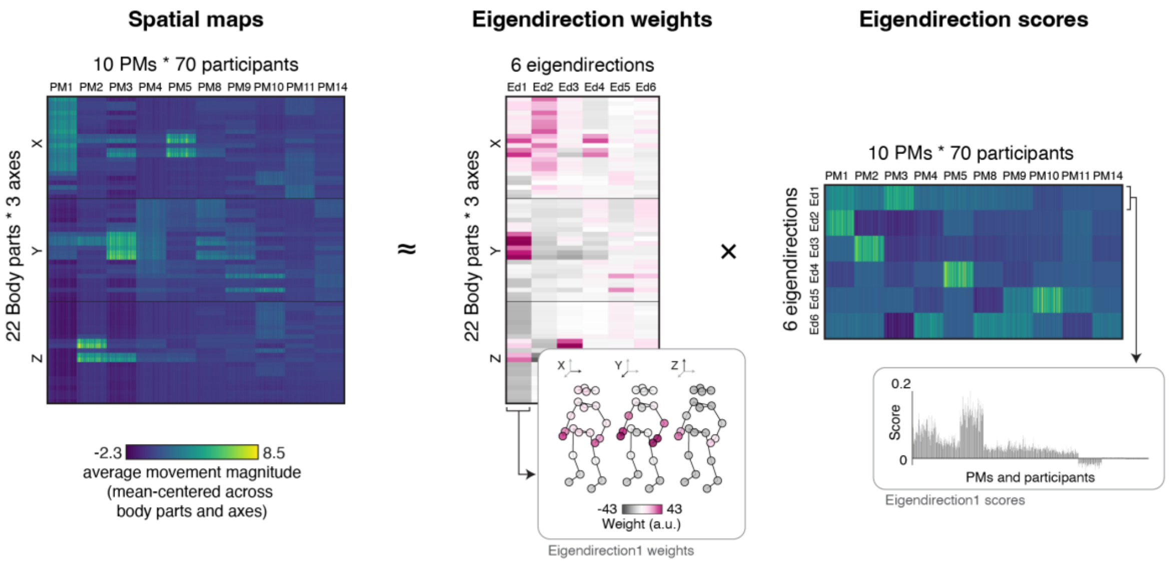

Figure S3. Extracting eigendirections of the Principal Movements (PMs) that were synchronized interpersonally. Related to Figures 3 & 4. Description of the second-layer PCA used to extract primary directions of movement – "eigendirections" – that generalized across the 10 PMs of interest (i.e. those associated with either partner-driven, music-driven, or hybrid synchrony, see Figures 2A–C). The 3D spatial maps of every PM and participant (i.e. the average movement magnitude of each body part along the 3D axes; see STAR Methods for details) were pooled into a "spatial maps" matrix (22 body parts along the 3 axes × 10 PMs across the 70 participants; left). These spatial maps were mean-centred (i.e. to quantify magnitude deviations from the average magnitude, across body parts and along the 3D axes). Next, by applying PCA to this matrix, the spatial maps were decomposed into a reduced subset of eigendirections that explained most of the spatial variance across PMs and participants. The spatial map of each PM and participant can now be described by a combination of primary directions of movement (eigendirection "weights", middle; large positive or negative weights indicate a relatively high or low amount of movement, respectively) weighted by their respective "scores" (right).

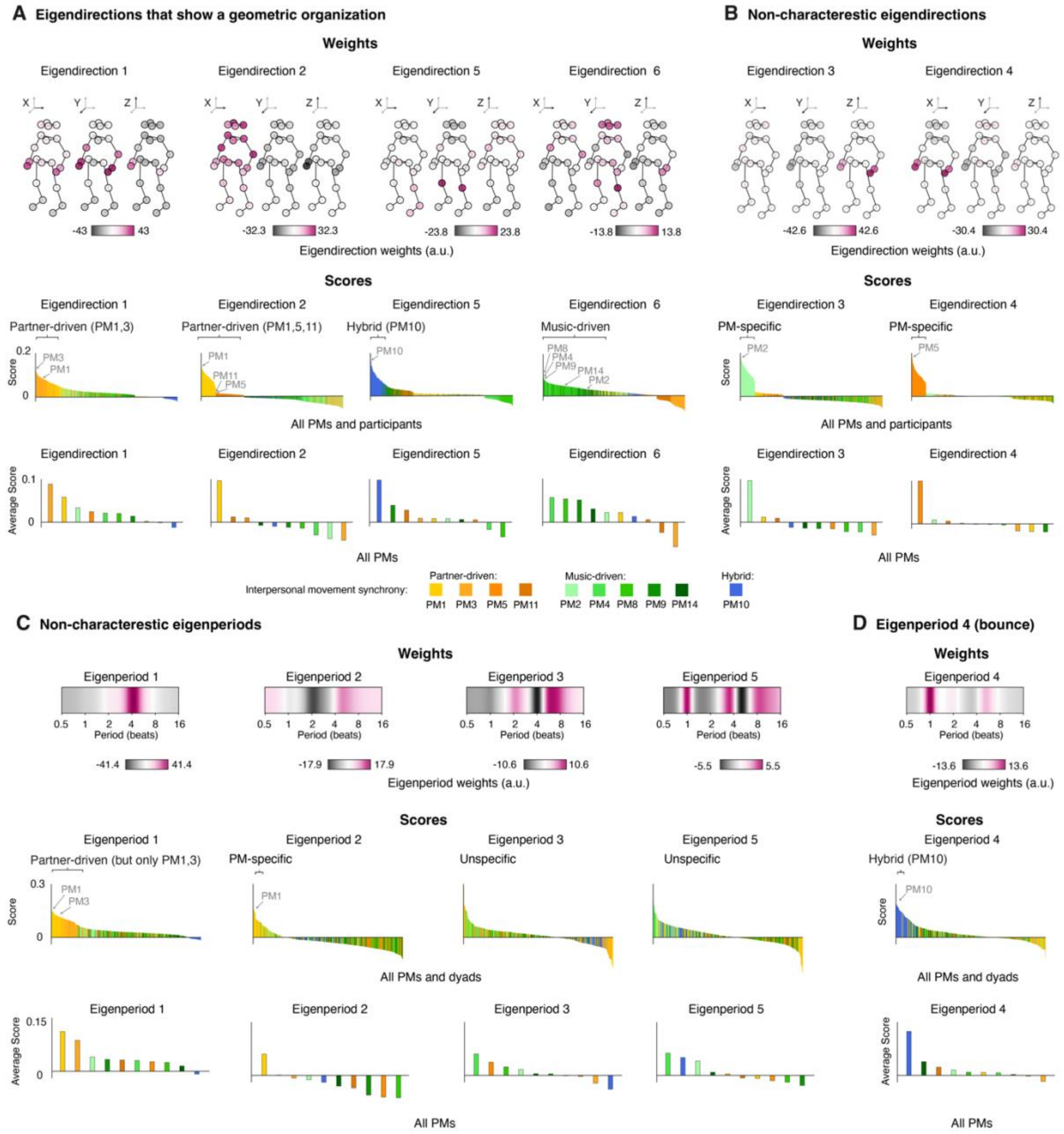

Figure S4. Characterization of the selected eigendirections and eigenperiods. Related to Figures 3 & 4. (A) Eigendirections revealing a geometric organization. **Top:** weights in 3D body-part coordinates, mean-centred. Large positive and negative weights indicate a relatively high and low amount of movement, respectively. **Bottom:** scores for all PMs and participants, as well as scores averaged across participants, ranked in descending order. These eigendirections show high scores for classes of PMs associated with either partner-driven (eigendirections 1 and 2), music-driven (eigendirection 6) or hybrid synchrony (eigendirection 5). **(B)** Same as A, but referring to non-characteristic eigendirections. These eigendirections are not characteristic of any class of PMs, but instead are specific for individual PMs. **(C)** Non-characteristic eigenperiods. **Top:** weights in period coordinates, mean-centred. Large positive and negative weights indicate a relatively high and low amount of synchrony, respectively. **Bottom:** scores for all PMs and dyads, as well as scores averaged across dyads, ranked in descending order. These eigendirections are not characteristic of any class of PMs, but instead are either PM-specific (eigenperiod 2), unspecific (eigenperiods 3 and 5) or only partially characteristic of partner-driven synchrony (eigenperiod 1). (**D**) Same as C, but referring to eigenperiod 4, characteristic of hybrid synchrony (i.e. synchrony of bounce).

Song	*Original title*<br>Artist	Genre	Tempo<br>(bpm [Hz])	Mean loudness<br>(LUFS)
1	*Get Down on It*<br>Kool and The Gang	Disco-funk	111.03 [1.85]	-10.9
2	*Treasure*<br>Bruno Mars	Disco-funk	116.07 [1.94]	-9.7
3	*Thriller*<br>Michael Jackson	Disco-funk	118.23 [1.97]	-9.8
4	*Dance with Somebody*<br>Whitney Houston	Disco-funk	118.95 [1.98]	-10.6
5	*Show Me Love*<br>Robin S	Electronic Dance Music	120.47 [2.01]	-9.9
6	*Call on Me*<br>Eric Prydz	Electronic Dance Music	125.93 [2.10]	-9.8
7	*We Found Love*<br>Rihanna	Electronic Dance Music	128.27 [2.14]	-9.5
8	*Freed from Desire*<br>Gala	Electronic Dance Music	129.06 [2.15]	-9.9

Table S1. Stimuli. Related to STAR Methods. Title and genre of the original songs that were rearranged. Tempo (in beats per minute (bpm) and Hz) and mean loudness (in Loudness Unit Full Scale (LUFS)) of the rearranged versions used in the experiment.

	Song 5	Song 6	Song 7	Song 8
Song 1	8.5%			
Song 2		8.5%		
Song 3			8.5%	
Song 4				8.5%

Table S2. Pairing of songs in the different-music condition. Related to STAR Methods. All pairs have the same relative tempo difference of 8.5%.
